# Aspiration, respiratory complications, and associated healthcare resource utilization among individuals with Rett syndrome

**DOI:** 10.1186/s13023-025-03757-6

**Published:** 2025-05-15

**Authors:** Nazia Rashid, Jonathan D. Darer, Charles Ruetsch, Xiaoyun Yang

**Affiliations:** 1 Medical Affairs, Acadia Pharmaceuticals, 3611 Valley Centre Dr STE 300, San Diego, CA 92130 USA; 2 Health Analytics, LLC, 6030 Daybreak Circle Suite 150 #351, Clarksville, MD 21029 USA

**Keywords:** Rett syndrome, Aspiration, Respiratory failure, Lower respiratory tract infection

## Abstract

**Background:**

Individuals with Rett syndrome (RTT) are at high risk for aspiration and also experience high rates of lower respiratory tract infections (LRTI) and respiratory failure (RF).

**Methods:**

A retrospective comparative cohort analysis was performed among 89 individuals with RTT with and without evidence of aspiration, using EHR structured and abstracted clinical notes data. Individuals with known or suspected aspiration (per clinical documentation) (cases) were compared to controls on aspiration risk factors, RF, LRTI, and hospitalization.

**Results:**

Of eligible individuals, 25 (28.1%) were aspiration cases. The cumulative rate of RF among RTT individuals with and without aspiration was 60.0% and 6.3%, respectively. Aspiration cases were more likely to have risk factors compared to controls during the 6-month baseline including epilepsy (54.5% vs. 4.5%), dysphagia (40.9% vs. 0%), GERD (31.8% vs. 0.0%), scoliosis (31.8% vs. 4.5%), and vomiting (18.2% vs. 0.0%). Aspiration cases were more likely to have LRTI (50% vs. 5.0%) and ≥ 1 inpatient admissions than non-aspiration controls (75.0% vs. 35.0%) (all *p* < 0.05).

**Conclusions:**

Individuals with RTT with known or suspected aspiration are at increased risk of LRTI, RF, and inpatient admissions. Providers should monitor aspiration and institute preventative measures among individuals with aspiration risk factors even in the absence of aspiration symptoms.

**Supplementary Information:**

The online version contains supplementary material available at 10.1186/s13023-025-03757-6.

## Background

Rett syndrome (RTT) is a rare neurodevelopmental disorder affecting 7.1 in 100,000 live female births and the leading cause of intellectual disability among young females [1, 2]. The diagnosis of RTT is made clinically, typically between ages 2–7 years old, by identification from the presence of four main criteria for typical RTT (loss of purposeful hand skills, loss of spoken language, gait abnormalities, stereotypic hand movement) or a combination of two main criteria and five supportive criteria (Supplemental Table 1) in atypical RTT [3]. Among the 85% of RTT cases with genetic testing identifies a mutation in MeCP2 (Methyl CpG Binding Protein 2), a gene involved in brain development and function [4]. In addition to cognitive and behavioral disabilities, individuals with RTT often have difficulties with mobility, feeding, and occurrence of seizures; creating substantial burden for family caregivers [5]. Many individuals with RTT can expect to live beyond age 40, however are constantly faced with multiple physical, muscular, and behavioral symptoms [6].

RTT syndrome is associated with multisystem comorbidities that contribute to overall healthcare utilization and mortality [7, 8]. The top three causes of mortality among individuals with RTT are respiratory conditions: LRTI (37%), aspiration and/or asphyxiation (32%), and respiratory failure (14%) [8]. Discoordination of breathing and swallowing among individuals with RTT often results with holding liquids in the pharynx, which increases the risk for aspiration [9]. The combination of highly prevalent breathing disturbances and dysphagia coupled with other risk factors for aspiration include gastrostomy placement, epilepsy, and scoliosis in RTT which increase the risk for aspiration and lower respiratory tract infections (LRTI) [10–12]. Dietary recommendations in RTT include the use of thickened liquids and purees to reduce risk for aspiration (Ramirez, et al., 2022). Aspiration events can often be silent or unwitnessed, along with frequent respiratory disturbances which can complicate the diagnosis process [13, 14].

Currently, there is limited published real-world data describing the rates of aspiration, respiratory complications and healthcare resource use (HCRU) among individuals with RTT. In this study, we utilized structured data (diagnosis/procedure codes) and unstructured data (clinical progress notes) from a Rett Center of Excellence located in the United States to better understand the prevalence of aspiration, respiratory complications, and related HCRU.

## Methods

### Data source

Structured electronic health record (EHR) data from Vanderbilt University Medical Center (VUMC) of diagnoses and procedure codes based on International Classification of Diseases, 9th and 10th Revision, Clinical Modification (ICD-9-CM, ICD-10-CM) and Current Procedural Terminology (CPT), 4th edition codes are included (Supplemental Table 2). In addition to diagnosis and procedure codes, data regarding symptoms and conditions was abstracted from clinical progress notes and swallowing studies by a team of clinicians using a structured abstraction tool with defined variables (Supplemental Tables 3, 4) and were utilized for this study to identify aspiration cases. Data for these analyses were licensed from Nashville Biosciences (www.nashville.bio) and the EHR structured dataset included outpatient encounters (including dates of service, provider specialty type from visit, encounter diagnoses), inpatient admissions (hospitalizations), emergency department (ED) visits, and orders (medications, laboratory tests, procedures, imaging exams). The VUMC is recognized as one of the Rett Center of Excellence facilities where they provide coordinated multidisciplinary care for children with RTT syndrome and RTT-related disorders. Data were de-identified and compliant with the Health Insurance Portability and Accountability Act; therefore, this study received an exemption from the Advarra Institutional Review Board.

### Study design

A retrospective, cohort study design was used to complete this chart and database analysis. The index date was defined as the date of the first aspiration event (case) observed by a diagnosis code from a medical encounter or abstracted from a clinical progress note or swallowing study report. RTT individuals who had an aspiration event were age-matched to those that did not have an aspiration event (control). The baseline period comprised the period up to 6 months prior to the index date and outcomes were evaluated during 12 months of follow up defined as post-index.

### Study population

**Fig. 1:**
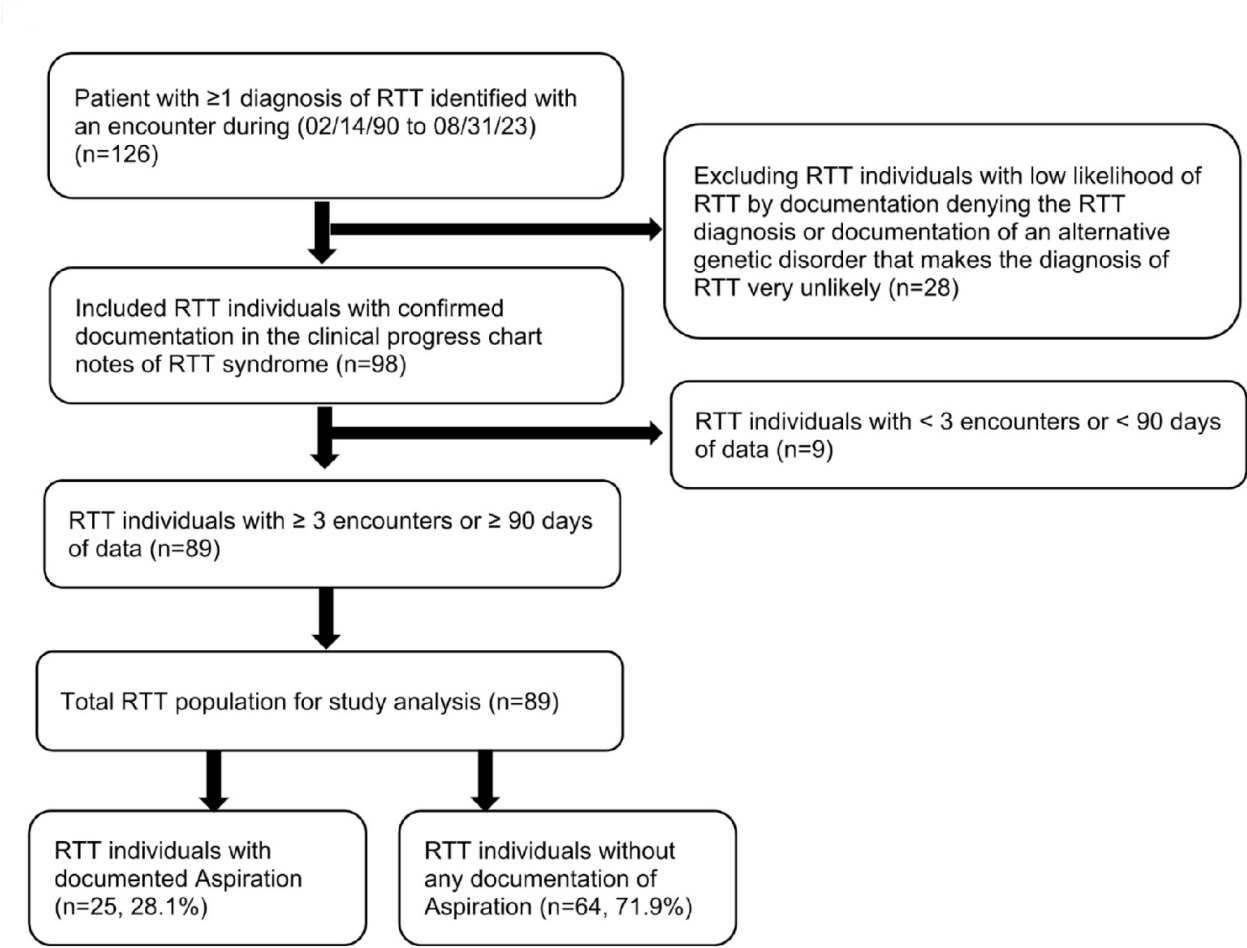
Attrition table and study groups for analyses

To be eligible for inclusion in this study, individuals met each of the following criteria during February 14, 1990 to August 31, 2023: a) ≥ 1 encounter (outpatient, inpatient, or emergency department) with a coded diagnosis of RTT (ICD-10 F82.4), b) a clinical progress note confirming the presence of RTT, c) ≥ 90 days of all-cause encounter history including time prior to and following the RTT diagnosis identified during our study time period, and d) ≥ 3 all-cause encounters with VUMC including encounters that occurred prior to and following the initial RTT diagnosis. RTT Individuals with a clinical progress note denying the presence of RTT were excluded from the study (Fig. 1).

To maximize the available sample of RTT individuals to assess risk factors of aspiration and respiratory outcomes, we performed three analyses: (a) evaluating the total RTT cohort identified within the VUMC [Total Patient Analysis], (b) evaluating baseline risk factors prior to first aspiration event [Aspiration Risk Factor Assessment], and (c) evaluating respiratory outcomes and HCRU following the first aspiration event [Respiratory Outcomes and HCRU Analysis]. RTT individuals were categorized into two groups for analyses b and c: aspiration and non-aspiration. An exploratory analysis was completed evaluating the cumulative rate of respiratory failure among total RTT individuals with aspiration versus the non-aspiration group.

### Study outcomes: aspiration event and non-aspiration group

Among the total RTT individuals identified in the study, an aspiration event (case) was determined by (a) presence of an ICD-9-CM or ICD-10-CM code for aspiration (507.0, 997.32, J69.0, J69.8, Y84.4) associated with an outpatient visit, inpatient admission, or ED visit among any submitted claim, or (b) presence of aspiration or suspected aspiration in a clinical progress note or a swallowing study. Because aspiration can be unwitnessed or silent, this study included RTT individuals with physician documentation of suspected aspiration as cases. RTT individuals without aspiration diagnosis codes or documentation of aspiration were placed in the non-aspiration group (control).

### Study measures

Covariate measures included patient race, patient sex, behavioral and psychiatric comorbidities (anxiety, behavior disorders), cardiac comorbidities (arrhythmias, QT prolongation), musculoskeletal comorbidities (scoliosis, kyphosis and other spinal deformities), neurologic comorbidities (epilepsy, sleep dysfunction, movement disorders, weakness/paralysis), gastrointestinal and nutrition comorbidities (constipation, dysphagia, gastrostomy, gastroesophageal reflux disease [GERD], nutritional deficiency, vomiting), and non-aspiration respiratory comorbidities (asthma, abnormal breathing, choking, apnea). Supplemental Table 2 includes the code list for these conditions.

The Risk Factor assessment evaluated the risk factors for aspiration documented in the baseline period. These risk factors included GI-specific conditions (dysphagia, GERD, vomiting, and gastrostomy), epilepsy, and scoliosis. The Respiratory Outcomes and HCRU Analysis were evaluated during the 12 months follow-up Period and evaluated recurrent aspiration events, LRTI, all-cause outpatient visits, all-cause ED visits, all-cause inpatient hospitalizations. Inpatient hospitalizations that included codes for aspiration, LRTI, and respiratory failure events were categorized as aspiration-specific, LRTI-specific, and respiratory failure-specific, respectively. Respiratory failure events were defined by encounters that included diagnosis codes for respiratory failure, acute respiratory distress syndrome, hypoxemia dependence on respirator, dependence upon supplemental oxygen, and respiratory arrest (Supplemental Table 2).

### Statistical analysis

Patient demographics, clinical characteristics, and outcome measures were reported descriptively as frequencies and percentages for categorical variables; mean, standard deviation (SD) for were reported for continuous variables. A cumulative time-to-event graph documenting respiratory failure events from birth was developed, comparing RTT individuals with and without an aspiration event. All analyses were performed using SAS version v9.4 (Cary, NC).

### Aspiration risk factor assessment

This analysis was limited to RTT individuals with an aspiration case who had ≥ 6 months of clinical history preceding the initial documented aspiration event and were 1:1 age-matched to controls (RTT individuals without an aspiration event). Eligible age-matched controls were also required to have ≥ 6 months of clinical history preceding the assigned index date. First, the age at index was identified for each RTT individual with an aspiration event. Second, the control was age-matched by the following method: (a) the age of the RTT individual with aspiration at index was matched to a RTT individual in non-aspiration group who was receiving care from VUMC around the same age as RTT aspiration case at index as defined by a the non-aspiration RTT individual age at clinical encounter within 2 years (+/-) of the age of RTT individual index aspiration event; (b) from this population, all individuals with ≥ 6 months of encounter history were retained; (c) one of the individual was selected at random, without replacement, from the pool of potential non-aspiration group for the matched control group. Bivariate analyses were performed comparing cases and controls regarding the percent of individuals with documentation of a risk factor in the baseline period.

### Respiratory outcomes and HCRU analysis

This analysis was limited to the RTT individuals with an aspiration event with a ≥ 12 months of clinical data (post-index) following the initial documented aspiration event and 1:1 matched to RTT individuals in the non-aspiration group. The RTT individual in the non-aspiration event was matched using the following method: (a) for each aspiration event, all non-aspirating individuals who had an encounter within +/-2 years from the aspiration index date were identified; (b) from this population, all individuals with ≥ 12 months of encounter history were retained; (c) one non-aspiration individual was selected at random, without replacement, from the pool of potential non-aspiration group. For an aspiration event individual, the assigned index date was the date of the closest encounter to the matched aspiration event age at index. The follow-up period was defined as the 12 months following the index date. Healthcare resource utilization was evaluated between the aspiration and non-aspiration groups.

## Results

A total of 126 individuals were identified with a RTT ICD-10 code. Of these, 28 individuals did not have a confirmatory RTT note and/or were excluded based on a clinical progress note denying the presence of RTT. An additional 9 individuals were excluded due to a lack of sufficient encounters or encounter history. RTT individuals identified with aspiration by ICD-10 code were also identified in clinical progress notes. An additional 14 patients were identified as aspiration by clinical progress notes and not by ICD-10 codes, which increased aspiration by 127% (Supplemental Table 1). A total of 89 individuals met study eligibility criteria and were included in the final population for analysis (Fig. 1).

**Fig. 2:**
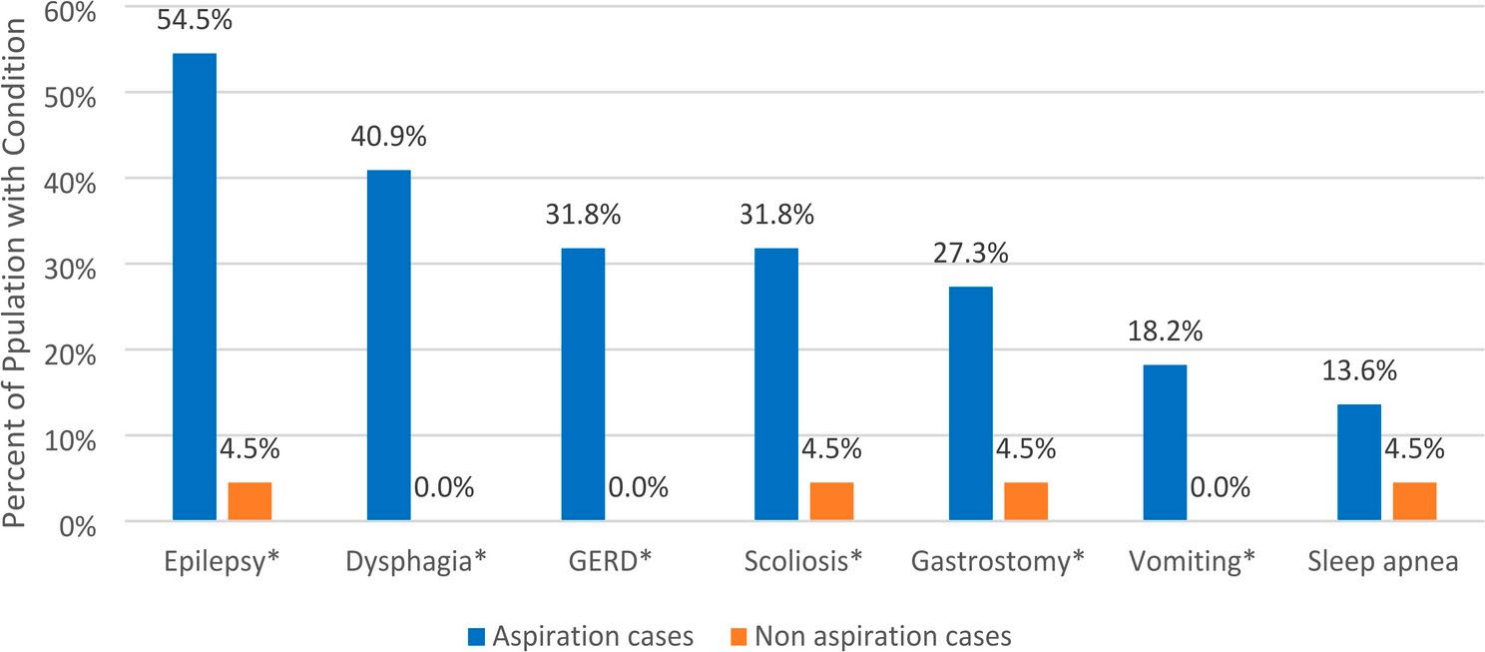
Aspiration risk factor analysis during 6-month baseline period. GERD = gastroesophageal reflux disease; *p-value < 0.05, statistically significant

**Fig. 3:**
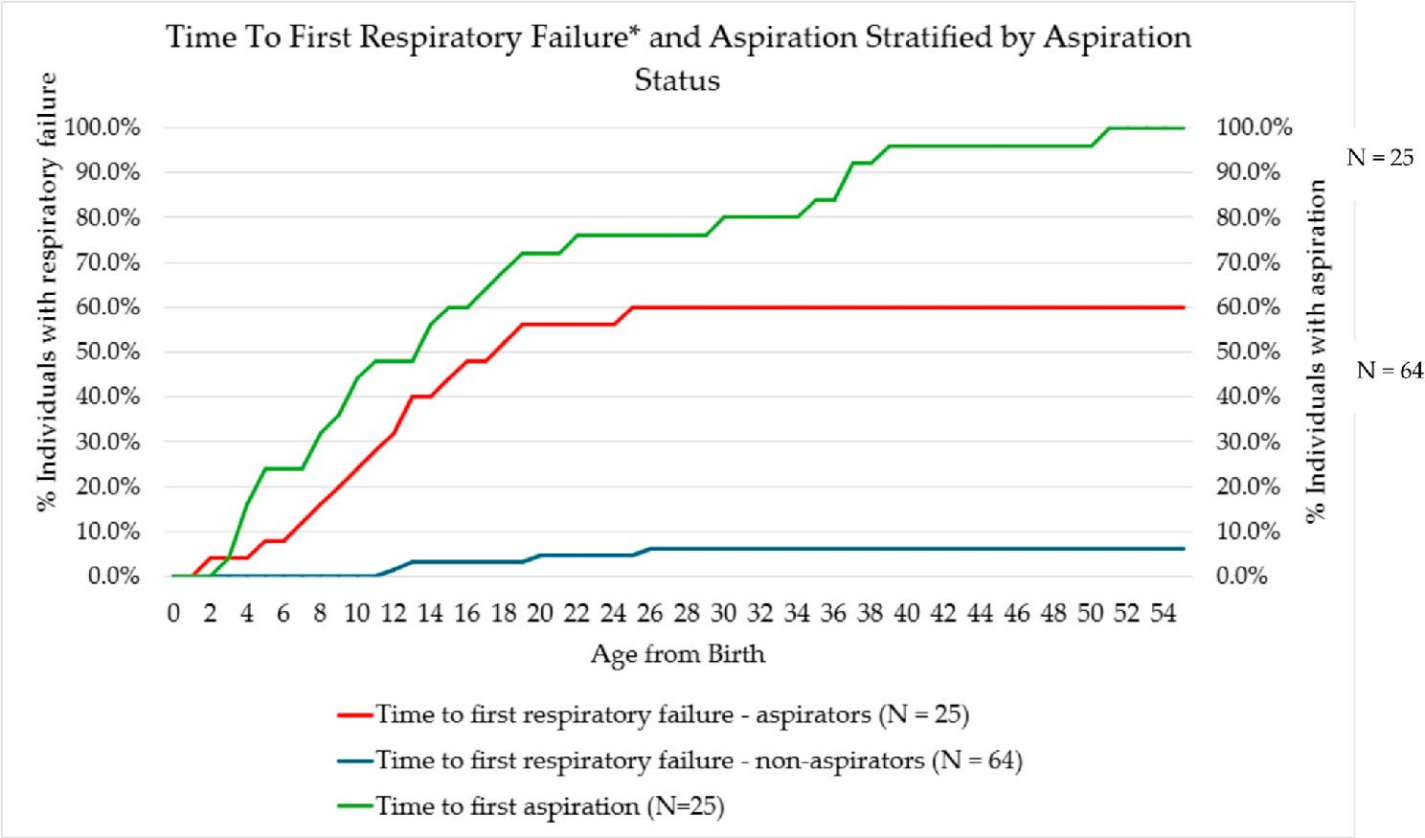
Cumulative rate of respiratory failure among total RTT individuals with aspiration vs. non-aspiration (*n* = 89). *Respiratory failure includes individuals diagnosed with hypoxemia and those who received mechanical ventilation

Of the 89 eligible individuals with RTT, 25 (28.1%) were identified as aspiration cases and 64 (71.9%) were non-aspiration controls (Fig. 1). The Total Patient Analysis identified 37 (41.5%) had a swallowing study to assess for aspiration. Of the 89 total RTT individuals, 82 (92.1%) were female, 59 (66.3%) were white, 6 (6.7%) were black/African American, 1 (1.1%) was Asian (Table 1). The most common aspiration risk factor was epilepsy (73.0%), followed by dysphagia (51.7%), GERD (44.9%), vomiting (40.4%), scoliosis (40.4%), gastrostomy (30.3%), and choking (23.6%) (Table 1). Other common comorbid conditions included constipation (70.8%), abnormal breathing patterns (61.8%), nutritional deficiency (40.4%), sleep dysfunction (36.0%), sleep apnea (32.6%), cardiac arrhythmias (23.6%), kyphosis and other spinal deformities (23.6%), anxiety (18%), behavioral disorders (18%), movement disorders (16.9%), and QT prolongation (13.5%) (Table 1). Prevalence of individuals with ≥ 1 inpatient admissions for respiratory conditions were respiratory failure (18.0%), LRTI (14.6%), and aspiration (9.0%) (Table 1). The cumulative rate of respiratory failure among the 25 RTT individuals with aspiration and 64 RTT individuals without aspiration is shown in Fig. 2. Over the full database of available data, RTT individuals with indication of aspiration were at greater risk to experience respiratory failure (60%) versus RTT individuals without indication of aspiration (6.3%) (Fig. 3).

**Table 1:** Demographic characteristics and select comorbidities among the total Rtt population (*n* = 89)

Patient and Clinical Characteristics	Total (*n* = 89)	%
Female	82	92.1%
Race		
Asian	1	1.1%
Black	6	6.7%
White	59	66.3%
Unknown	23	25.8%
Aspiration		
Aspiration (known or suspected)	25	28.1%
Non-aspiration	64	71.9%
Abnormal breathing and other respiratory conditions		
Asthma	11	12.4%
Abnormal breathing	55	61.8%
Choking**	21	23.6%
Sleep apnea	29	32.6%
Behavioral and psychiatric comorbidities		
Anxiety	16	18.0%
Behavior disorders (e.g., aggression)	16	18.0%
Cardiac comorbidities		
Cardiac arrhythmias	21	23.6%
QT prolongation	12	13.5%
Gastrointestinal and nutritional comorbidities*		
Constipation	63	70.8%
Dysphagia	46	51.7%
Gastrostomy	27	30.3%
GERD	40	44.9%
Nutritional deficiency	39	43.8%
Vomiting	36	40.4%
Musculoskeletal comorbidities		
Scoliosis	36	40.4%
Kyphosis and other spinal deformities	21	23.6%
Neurologic comorbidities		
Epilepsy	65	73.0%
Sleep dysfunction	32	36.0%
Movement disorders	15	16.9%
Weakness/Paralysis	32	36.0%
≥ 1 Inpatient admissions		
Aspiration-specific	8	9.0%
LRTI-specific	10	14.6%
Respiratory failure-specific	16	18.0%

*GI clinical progress notes were used to identify more RTT individuals with these comorbidities versus diagnoses codes alone. LRTI = lower respiratory tract infection; GERD = gastroesophageal reflux disease; ** choking was seen in clinical notes only

### Aspiration risk factors analysis

The 22 eligible aspiration cases were significantly more likely to have documented risk factors during the 6-month baseline period than non-aspiration controls including epilepsy (54.5% vs. 4.5%, *p* < 0.05), dysphagia (40.9% vs. 0%, *p* < 0.05), GERD (31.8% vs. 0.0%, *p* < 0.05), scoliosis (31.8% vs. 4.5%, *p* < 0.05), gastrostomy (27.3% vs. 4.5%, *p* < 0.05), and vomiting (18.2% vs. 0.0%, *p* < 0.05) (Fig. 2).

### Respiratory outcomes and HCRU analysis

The 20 eligible aspiration cases were significantly more likely to have LRTI than non-aspiration controls (50% vs. 5.0%, *p* < 0.05). Almost a third of individuals (30%) with aspiration had recurrent aspiration in the following 12 months. Aspiration cases were also significantly twice more likely to have ≥ 1 inpatient admissions for any reason than non-aspiration controls (75.0% vs. 35.0%, *p* < 0.05). Rates of all-cause outpatient visits and ED visits were similar among the two groups; however, the number of inpatient admissions were higher for the aspiration cases vs. non-aspirating controls (9.4 vs. 2.6, ns), (Table 2).

**Table 2:** Respiratory outcomes and healthcare resource utilization during 12-month follow up period

Outcomes during 12-month follow up period	Aspiration Cases (*n* = 20)		Non-Aspiration Controls (*n* = 20)		*p*-value
Aspiration, n (%)	6	(30.0)	0	(0.0)	-
Lower respiratory tract infection, n (%)	10	(50.0)	1	(5.0)	< 0.05
All-cause outpatient visits, n (%)	18	(90.0)	18	(90.0)	ns
Number of all-cause OP visits (median, q1-q3)	9.0	5.0–15.0	4.5	3.0–7.0	ns
All-cause emergency department visits, n (%)	3	(15.0)	4	(20.0)	ns
Number of all-cause ED visits, (median, q1 - q3)	1.0	1.0–4.0	1.0	1.0–2.0	ns
All-cause inpatient admissions, n (%)	15	(75.0)	7	(35.0)	< 0.05
Number of all-cause IP admissions, (median, q1 - q3)	6.0	1.0–18.0	2.0	1.0–4.0	ns

ED = emergency department; IP-inpatient; OP = outpatient; SD = standard deviation; ns = non-significant

## Discussion

To better understand the onset and complications of aspiration in individuals with RTT, the study analyzed a combination of structured EHR data, unstructured data extracted from clinical progress notes and swallowing studies from VUMC, a RTT Center of Excellence facility. The clinical progress notes were essential for this study because diagnosis codes alone do not provide the true prevalence of the outcome studied. The results demonstrated high prevalence of risk factors for aspiration including dysphagia, epilepsy, scoliosis, GERD, and vomiting. Risk factors were more commonly documented in aspiration cases versus matched non-aspiration controls during the 6 months preceding aspiration (Aspiration Risk Factor Assessment). Individuals with aspiration also were more likely to have ≥ 1 inpatient admissions after aspiration than matched controls (Table 2).

Aspiration can lead to a broad spectrum of pulmonary diseases such as airway obstruction, pneumonia, chemical pneumonitis, or acute respiratory distress syndrome with significant morbidity and mortality [15]. Prior published research has shown individuals with RTT most commonly die of LRTI, aspiration/asphyxiation, and respiratory failure [8]. The mechanism by which individuals with RTT develop severe respiratory illness has not been well described. While breathing disorders such breath holding and hyperventilation, are common in RTT and have been attributed to autonomic and/or brain stem dysfunction, it is not clear that they would result in LRTI or frank respiratory failure [10]. Individuals with RTT also have also documented lung changes starting at a relatively early age including centrilobular nodules (66.7%), bronchiolectasis (60%), and ground-glass opacities (26.7%), associated with chronic [16, 17]. As such, the conclusion that aspiration is often the cause of respiratory distress in individuals with RTT would appear logical.

Documenting aspiration events and aspiration-related illness (i.e., pneumonitis, pneumonia) can be challenging, and the true prevalence of aspiration and related respiratory complications is not known [15]. Aspiration can be a silent event, affecting over 26% of children with dysphagia [13, 18]. Aspiration associated with swallowing dysfunction can be diagnosed via swallowing studies [19], yet a negative swallowing study does not rule out aspiration indefinitely. Individuals with epilepsy with tonic-clonic seizures may have normal swallowing but be unable to protect their airway during the peri-seizure period [20, 21]. Gastrostomy placement is an effective treatment to address concerns with feeding, malnutrition, and swallowing dysfunction and the published estimate of gastrostomy placement in individuals with RTT is estimated at 28%, very similar to the findings of this study at 30% [22]. The placement of a gastrostomy, unfortunately, appears to increase risk for aspiration and LRTI, mostly likely because of gastroesophageal dysmotility and GERD [12].

During the study period, RTT individuals were not on any FDA approved treatments for RTT and management has traditionally been focused upon symptom reduction and functional improvement. While documenting aspiration events may be difficult, identifying individuals at high-risk for aspiration and providing anticipatory management would seem to be prudent, though consensus guidelines are relatively silent on the subject (Fu, et al., 2020). Three-quarters of individuals with aspiration experience ≥ 1 inpatient admissions in the 12-month period post-aspiration. The high rate of healthcare utilization highlights the morbidity of this subpopulation and the need for more effective diagnostic and treatment to avoid aspiration and its consequences.

RTT syndrome is a rare disease and identifying cohorts for real-world evidence generation is difficult. By extracting data from clinical notes, we were able to identify RTT population with greater accuracy and enhanced the prevalence of important conditions and symptoms versus coded data alone. Using a combination of coded data and information extracted from the clinical narrative can enable researchers to provide a more robust and accurate picture of the patient journey.

Strengths of this study include the real-world evidence generated for healthcare providers who care for individuals with RTT and provide insightful data of aspiration among individuals with RTT at a Rett Center of Excellence. The combination of structured and unstructured data appears to address deficiencies associated with structured data alone, substantially increasing symptom prevalence. In addition, the ability to age-match comparator populations provides more compelling evidence related to both the dramatic differences in the prevalence of pre-aspiration risk factors and for the post-aspiration respiratory and hospitalization outcomes. There are also limitations to address, such as this study was performed based upon data derived from VUMC, a RTT Center of Excellence, and their population of RTT individuals may differ from other health systems. This study was limited to clinical encounters and documentation from VUMC and may not represent the full healthcare experience. Individuals may be experiencing symptoms at home or taking over-the-counter medications (such as proton pump inhibitors which are a risk factor for LRTI) which are not documented in the EHR. In addition, the population of VUMC individuals with RTT is relatively small and has substantial variability in available data, limiting the ability of this study to detect significant differences between cases and comparators or perform meaningful multivariate analyses. RTT syndrome is also a heterogeneous condition with typical and atypical variants and can be associated with different genetic mutations. This study did not seek to address different RTT phenotypes and further research is needed to understand aspiration in different subpopulations.

## Conclusion

Individuals with Rett syndrome have many comorbidities (i.e., dysphagia, scoliosis, GERD, choking, and epilepsy) that are also risk factors for aspiration. Individuals with documented known or suspected aspiration are at increased risk of LRTI, respiratory failure, and increased inpatient admissions. Providers should monitor individuals with RTT for aspiration and institute preventative measures among individuals with aspiration risk factors even in the absence of aspiration symptoms.

## Electronic supplementary material

Below is the link to the electronic supplementary material.

Supplementary Material 1

## Data Availability

The data that support the findings of this study are available from Nashville Biosciences but restrictions apply to the availability of these data, which were used under license for the current study, and so are not publicly available. Data are however available with permission from Acadia Pharmaceuticals.
