# Supplementary material for: Aspiration, respiratory complications, and associated healthcare resource utilization among individuals with Rett syndrome
Source: Orphanet J Rare Dis. 2025 May 15;20:232. doi: 10.1186/s13023-025-03757-6 (PMC12082938; doi:10.1186/s13023-025-03757-6)

**Supplementary Materials**

**Supplemental Table 1. Supportive Criteria for atypical RTT^1^**

1 Breathing disturbances when awake

2 Bruxism when awake

3 Impaired sleep pattern

4 Abnormal muscle tone

5 Peripheral vasomotor disturbances

6 Scoliosis/kyphosis

7 Growth retardation

8 Small cold hands and feet

9 Inappropriate laughing/screaming spells

10 Diminished response to pain

11 Intense eye communication - “eye pointing”

^1^Neul et al., 2010

**Supplemental Table 2. Mapped Clinical Domains, Diagnosis Descriptions, and Associated Codes**

| **Clinical Domain** | **Diagnosis Description** | **ICD-9, ICD-10, and CPT Codes** |
| --- | --- | --- |
| Aspiration | Pneumonitis due to inhalation | 507.X, 997.32, J69.0, J69.8 |
| Aspiration | Aspiration of fluid as the cause of abnormal reaction of the patient | 997.32, Y84.4 |
| Abnormal breathing and other respiratory conditions | Dyspnea | 786.05, R06.0X |
| Abnormal breathing and other respiratory conditions | Stridor | 786.1, R06.1 |
| Abnormal breathing and other respiratory conditions | Wheezing | 786.07, R06.2 |
| Abnormal breathing and other respiratory conditions | Periodic breathing | 786.04, R06.3 |
| Abnormal breathing and other respiratory conditions | Hyperventilation | 786.01, R06.4 |
| Abnormal breathing and other respiratory conditions | Apnea | 786.03, R08.81, P28.4X |
| Abnormal breathing and other respiratory conditions | Tachypnea | 786.07, R08.82 |
| Abnormal breathing and other respiratory conditions | Abnormal breathing - Other | 786.09, R08.89, R06.89 |
| Abnormal breathing and other respiratory conditions | Abnormal breathing – Unspecified | 786.00, R08.9 |
| Abnormal breathing and other respiratory conditions | Asthma | 493.XX, J45.XX, 518.3 |
| Abnormal breathing and other respiratory conditions | Sleep apnea | 327.2X, 780.51, 780.53, 780.57, G47.3X |
| Behavioral and Psychiatric | Anxiety | 300.0X, F06.4, F41.X |
| Behavioral and Psychiatric | Behavioral disorders (e.g., aggression) | 294.11, 312.X, F90.X, F91.X, F94.X, F95.X, F98.XX, R45.1, R45.4-R45.6, R45.83 |
| Cardiac | Cardiac arrhythmias | I49.9, 427.9, 427.89 |
| Cardiac | QT Prolongation | I45.81, 426.82 |
| GI and Nutrition | Dysphagia | R13.1X, 787.2X |
| GI and Nutrition | Gastrostomy | 536.4X, Z93.1, K94.2X |
| GI and Nutrition | Vomiting and hematemesis | 787.01, 787.03, 933.1, R11.1X, R11.2, 578.0, K92.0 |
| GI and Nutrition | Constipation | K59.00, K59.01, K59.04, K59.09, 564.00, 564.01, 564.09 |
| GI and Nutrition | GERD | 530.81, 530.11, K21.XX |
| GI and Nutrition | Nutritional deficiency | 26X.XX, 783.22, 783.41, 783.7, E40, E41, E42, E43, E44, E45, E46, E63.X, R62.51, R62.7 |
| LRTI | Viral bronchitis | J20.3-J20.7, J21.0-J21.1 |
| LRTI | Acute bronchitis | 466.0, J20.X |
| LRTI | Bronchiolitis | 466.1X, J21.X |
| LRTI | Viral pneumonia | 480.X, J09.X1, J10.0X, J12.X |
| LRTI | Bacterial pneumonia | 481.X, 482.X, J13.X, J14.X, J15.X |
| LRTI | Pneumonia due to other infectious organisms, not elsewhere classified | 483.X, J16.X |
| LRTI | Pneumonia in diseases classified elsewhere | 484.X, J17.X |
| LRTI | Pneumonia, organism unspecified | 483.X, J18.X |
| LRTI | Lower respiratory tract infection | J22.X |
| LRTI | Bronchitis, not specified as acute or chronic | J40.X |
| Musculoskeletal | Scoliosis | 737.3X, 737.43, M41.XX |
| Musculoskeletal | Kyphosis and other spinal deformities | R29.3, M43.9, M40.05, M40.204, Q67.5, M40.14, 754.2, M40.57, M40.209, Q76.414, 737.29, 781.92 |
| Neurologic | Epilepsy | 345.X, G40.xx |
| Neurologic | Sleep dysfunction | 307.44, 327.8, 780.59, G47.0x-G47.2x, G47.4-G47.6, G47.8, G47.9, F51.01, F51.04 |
| Neurologic | Movement disorders | 781, G25.5, G26, G25.9, G47.61, R25.8 |
| Neurologic | Weakness or paralysis | M62.81, 728.87, G82.50, 343.2, P94.2, 342.11, 342.12, 334.1 |
| Respiratory failure | Acute respiratory distress syndrome | J80 |
| Respiratory failure | Respiratory failure | 518.5X, 518.8X, J96.XX, J95.82X |
| Respiratory failure | Dependence on respirator | Z99.11, V46.1X |
| Respiratory failure | Dependence upon supplemental oxygen | V46.2, Z99.81 |
| Respiratory failure | Respiratory arrest | 799.1, R09.2 |
| Respiratory failure | Hypoxemia | 799.02, R09.02 |
| Swallowing studies |  | 92610, 74230 |

GI = gastrointestinal; LRTI = lower respiratory tract infection

**Supplemental Table 3. Data Abstraction Guide for NashBio for Aspiration and Related Concepts.**

| **Source Document** | **Variable name** | **Variable responses** |
| --- | --- | --- |
| Most informative aspiration or respiratory condition-related clinical progress note | History of breathing disturbance or respiratory symptom | Type of breathing disturbance or respiratory symptom   - Dyspnea - Orthopnea - Breath holding or apnea while awake - Tachypnea - Hyperventilation - Stridor - Wheezing - Cough |
| Most informative gastrointestinal related clinical progress note | Gastrointestinal risk factor for aspiration | - Dysphagia - GERD - Gastrostomy - Vomiting |
| Most informative aspiration or respiratory condition-related clinical progress note | History of choking or asphyxiation | - Choking - Asphyxiation |
| Most informative aspiration or respiratory condition-related clinical progress note | Diagnosis or suspected diagnosis of aspiration, aspiration pneumonitis, or aspiration pneumonia | - Aspiration - Aspiration pneumonitis - Aspiration pneumonia |
| Most informative aspiration or respiratory condition-related clinical progress note | Diagnosis of Lower Respiratory Tract Infection | - Pneumonia - Bronchitis |
| Most informative aspiration or respiratory condition-related clinical progress note | Diagnosis of Respiratory Failure | - Respiratory failure - Hypoxia or hypoxemia |
| Most informative aspiration or respiratory condition-related clinical progress note | History of respiratory support | - Use of oxygen - Use of CPAP - Use of BIPAP - Use of other mechanical ventilation |
| Narrative results of swallowing study (e.g., bedside swallowing study, videofluoroscopic swallow study, fiberoptic endoscopic evaluation of swallowing | Aspiration present | - Yes - No |

**Supplemental Table 4. Variable Extraction for Reporting from NashBio Clinical Progress Notes.**

| **Domain** | **Variable Details** | **Variable Format** |
| --- | --- | --- |
| Aspiration and Respiratory Issues | Symptom or condition names | String/Character |
|  | Known or suspected | Categorical |
|  | Date of onset | Date |
| Provider | Provider type/specialty | String/Character |
|  | Date of clinical progress notes written | Date |
| Swallowing Study | Swallowing study name | String/Character |
|  | Swallowing study date | Date |
|  | Swallowing study result (Aspiration present; Aspiration not present) | Categorical |

**Supplemental Fig. 1. Identification of Individuals with Aspiration**


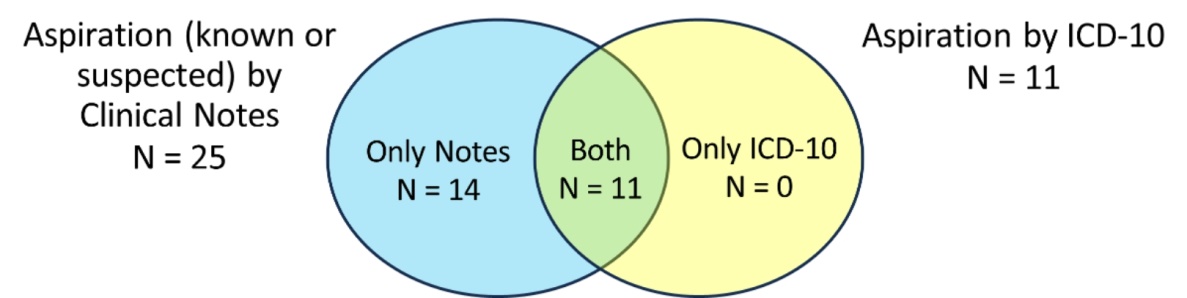

Supplement: Supplementary file 1 — Supplementary Material 1 [file 13023_2025_3757_MOESM1_ESM.docx]
